# Gray matter volume alterations in patients with strabismus and amblyopia: voxel-based morphometry study

**DOI:** 10.1038/s41598-021-04184-w

**Published:** 2022-01-10

**Authors:** Ting Su, Pei-Wen Zhu, Biao Li, Wen-Qing Shi, Qi Lin, Qing Yuan, Nan Jiang, Chong-Gang Pei, Yi Shao

**Affiliations:** 1 Fujian Provincial Key Laboratory of Ophthalmology and Visual Science, Eye Institute of Xiamen University, School of Medicine, Xiamen University, Xiamen, Fujian 361102 People’s Republic of China; 2 Department of Ophthalmology, Massachusetts Eye and Ear, Harvard Medical School, Boston, MA 02114 USA; 3 Department of Ophthalmology, The First Affiliated Hospital of Nanchang University, Nanchang, Jiangxi 330006 People’s Republic of China

**Keywords:** Diagnostic markers, Eye diseases

## Abstract

This study proposes the use of the voxel-based morphometry (VBM) technique to investigate structural alterations of the cerebral cortex in patients with strabismus and amblyopia (SA). Sixteen patients with SA and sixteen healthy controls (HCs) underwent magnetic resonance imaging. Original whole brain images were analyzed using the VBM method. Pearson correlation analysis was performed to evaluate the relationship between mean gray matter volume (GMV) and clinical manifestations. Receiver operating characteristic (ROC) curve analysis was applied to classify the mean GMV values of the SA group and HCs. Compared with the HCs, GMV values in the SA group showed a significant difference in the right superior temporal gyrus, posterior and anterior lobes of the cerebellum, bilateral parahippocampal gyrus, and left anterior cingulate cortex. The mean GMV value in the right superior temporal gyrus, posterior and anterior lobes of the cerebellum, and bilateral parahippocampal gyrus were negatively correlated with the angle of strabismus. The ROC curve analysis of each cerebral region confirmed the accuracy of the area under the curve. Patients with SA have reduced GMV values in some brain regions. These findings might help to reveal the potential pathogenesis of SA and its relationship with the atrophy of specific regions of the brain.

## Introduction

Strabismus is a common visual developmental disease due to dysfunction of the extraocular muscles, which is thought to be associated with a deformity in the cerebral visual pathway that intermediates ocular movement^1^, and leads to the lesion of stereopsis and binocularity^2^. The prevalence of adult new-onset strabismus is reportedly 54.1 cases per 100,000 people^3^, and strabismus is often correlated with amblyopia^4^. Amblyopia is an optic disorder that is defined as a decline in visual acuity (VA) resulting from abnormal binocular development^5^. Amblyopia is likely to contribute to perceptual strabismus, and, conversely, strabismus might exacerbate amblyopia.

In recent years, magnetic resonance imaging (MRI) has been increasingly developed, and offers a noninvasive imaging approach to determine both functional and structural alterations in the cerebrum^6^. Some investigators have used various MRI analytical techniques to detect intrinsic activation of brain regions in amblyopic and strabismic patients, such as amplitude of low-frequency fluctuation (ALFF), degree centrality (DC), and regional homogeneity (ReHo) These techniques facilitate the investigation of spontaneous activities of the brain and reveal the underlying mechanisms occurring in the diseased eye^7–9^. In our previous studies, we used the ALFF method to explore alterations in cerebral activity in participants with strabismus and amblyopia (SA)^10^. We observed functional cerebral alterations in subjects with SA; however, morphological differences in the neuromechanism in the brain of patients with SA remained unclear.

Voxel-based morphometry (VBM) is a broadly applied technique used to analyze cerebral alterations at the voxel level, which can reveal the anatomical changes in whole brain tissue by quantitatively evaluating the density, volume, and distribution of gray matter and white matter across several cerebral regions^11^. Recently, the VBM approach has been widely utilized for the diagnostic, therapeutic, and prognostic assessment of many ocular diseases, such as cataract, glaucoma, and optic neuritis^12–14^. The technique has been successfully proven to be reliable and credible in identifying abnormal anatomy in ophthalmological disorders and conducive to elucidate the pathophysiological mechanisms. However, previous VBM studies have only focused on either strabismic or amblyopic patients^15–17^. The present study aims to use the VBM method to explore differences in gray matter and white matter between patients with SA and healthy controls (HCs), and to investigate their relationship with clinical features.

## Methods

### Subjects

Sixteen patients with SA (five males and eleven females) were enrolled in the Ophthalmology Department of the First Affiliated Hospital of Nanchang University. Prism and cover test measurements were performed to measure the angle of strabismus. We formulated the inclusion criteria as follows: (1) patients with strabismus; (2) the differences in the best-corrected VA (≥ 0.20 logMAR units) between two eyes were greater than one line; (3) presence of central fixation; (4) no other eye diseases. Patients who met any of the following criteria were excluded: (1) previous ocular surgery; (2) patients with systemic disease, such as psychiatric disorders or cerebral infarction; (3) drug or alcohol addiction; (4) unable to undergo an MRI scan.

Sixteen HCs (five males and eleven females), similarly matched in age and sex with the patients with SA, were enlisted. Each HC conformed to the following standards: (1) no history of ophthalmological disease, with best-corrected VA ≤ 0 logMAR units; (2) absence of psychiatric disorders or malformations in the cerebral parenchyma; (3) capable of undergoing MRI examination.

This study was approved by the Medical Ethics Committee of the First Affiliated Hospital of Nanchang University. The procedures were in accordance with the principles of the Declaration of Helsinki. All participants volunteered to cooperate and signed informed consent forms, with awareness of the study purposes and potential risks.

### MRI parameters

All participants were scanned with a 3-Tesla MRI scanner (Trio, Siemens, Munich, Germany). High-resolution T1-weighted images with a magnetization-prepared rapid gradient echo (MP-RAGE) sequence was obtained. The following comprised the specific scanning parameters: 176 images of 1 mm section thickness; echo time = 2.26 ms; repetition time = 1900 ms; field of view = 215 × 230 mm; acquisition matrix = 256 × 256; flip angle = 9°.

### VBM analysis

We preprocessed the structural image data using the voxel-based morphometry toolbox (VBM8, http://dbm.neuro.uni-jena.de/vbm8/), Statistical Parametric Mapping (SPM8, http://www.fil.ion.ucl.ac.uk), and MATLAB 7.9.0 software (R2009b; The Mathworks, Inc, Natick, MA, USA). The brains were divided into three parts as gray matter, white matter and cerebrospinal fluid using the default estimation options on VBM8, as previously described^18^. The preprocessed data were then standardized to conform to the Montreal Neurological Institute (MNI) criteria. The generated template produced by DARTEL (Diffeomorphic Anatomical Registration Through Exponentiated Lie Algebra) analysis was used to standardize the white matter and gray matter of subjects. The modulated volumes were then smoothed with a Gaussian kernel of 6-mm full width at half maximum.

### Statistical analysis

The SPSS 20.0 software (SPSS, IBM Corporation, NY, USA) was used to compare the differences in clinical data between patients with SA and HCs. In addition, the independent sample *t*-test (age and best-corrected VA) and chi-squared test (sex) were applied. A *p* value < 0.05 was set as statistically significant.

The differences in gray matter volume (GMV) and white matter volume (WMV) between the SA and HC groups were analyzed with the SPM8 toolkit using general linear model (GLM) analysis. The level of significance was considered to be *p* < 0.01.

Receiver operating characteristic (ROC) curve analysis was applied to distinguish the mean GMV values in different cerebral areas of the patients with SA from those of HCs.

### Brain-behavior analysis

The mean GMV and WMV values were calculated by averaging every GMV or WMV value over entire voxels for each regions of difference (RODs) according to the VBM measuring results, using the Resting-State fMRI Data Analysis Toolkit (REST) (http://www.restfmri.net). Correlation analysis was applied to explore the relationship between the mean GMV value of specific cerebral regions and clinical manifestations in the SA group. A *p* value < 0.01 was deemed to indicate a statistically significant difference.

### Ethics approval

This study was approved by the Medical Ethics Committee of the First Affiliated Hospital of Nanchang University. The procedures were in accordance with the principles of the Declaration of Helsinki.

### Informed consent

Informed consent was obtained from all individual participants included in the study, with awareness of the study purposes and potential risks.

## Results

### Demographics and visual measurements

No statistical differences were noted in age (*p* = 0.626) or best-corrected VA of the fellow eye (*p* = 0.847) between subjects with SA and HCs. The best-corrected VA of the amblyopic eye in the SA group was significantly higher than that in the HC group (*p* < 0.001) (details are presented in Tables 1).

**Table 1:** Demographics and clinical measurements of SA and HC groups.

	SA	HC	t-value	*p* value
Male/female	5/11	5/11	N/A	> 0.99
Age (years)	24.38 ± 6.03	25.69 ± 5.83	0.626	0.536
Handedness	16 R	16 R	N/A	> 0.99
Duration (years)	18.06 ± 9.87	N/A	N/A	N/A
Esotropia/exotropia	5/11	N/A	N/A	N/A
Sensory strabismus/alternating strabismus	8/8	N/A	N/A	N/A
Angle of strabismus (PD)	28.44 ± 11.79	N/A	N/A	N/A
Best-corrected VA-AE (logMAR)	0.78 ± 0.52	− 0.05 ± 0.08	6.274	< 0.001
Best-corrected VA-FE (logMAR)	− 0.01 ± 0.11	− 0.01 ± 0.07	− 0.195	0.847

Independent t-tests comparing the two groups (*p* < 0.05 represented statistically significant differences). Data shown as mean standard deviation or number.

*SA* strabismus with amblyopia, *HC* healthy control, *N/A* not applicable, *PD* prism diopter, *VA* visual acuity, *AE* amblyopic eye, *FE* fellow eye, *R* right.

### VBM differences

**Figure 1:**
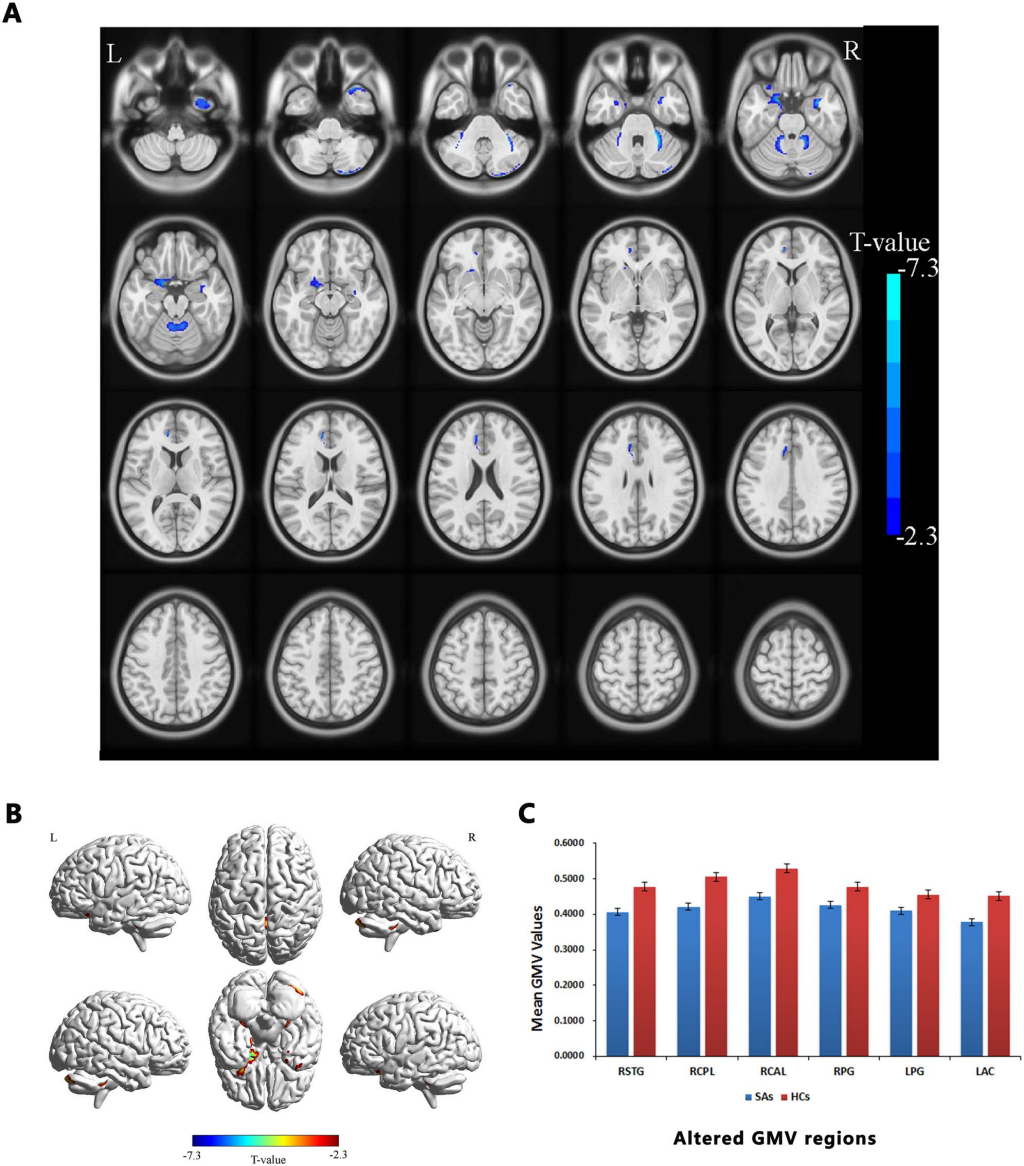
Voxel-wise comparison of GMV in the SA and HC group. *Notes*: (**A**,**B**) Significant differences in GMV were observed. The blue regions denote lower GMV values. (**C**) The mean GMV values between the SA and HC groups. *GMV* gray matter volume, *SA* strabismus with amblyopia, *HC* healthy control, *RSTG* right superior temporal gyrus, *RCPL* right posterior lobe of cerebellum, *RCAL* right anterior lobe of cerebellum, *RPG* right parahippocampal gyrus, *LPG* left parahippocampal gyrus, *LAC* left anterior cingulate.

Compared with the HCs, GMV values in the SA group showed significant reduction in the right superior temporal gyrus, posterior and anterior lobes of the cerebellum, bilateral parahippocampal gyrus, and left anterior cingulate cortex (Fig. 1 and Table 2). However, there were no statistically significant differences in mean values of whole brain GMV and WMV between two groups (Table 3).

**Table 2:** Brain areas with significantly group GMV differences between groups.

Conditions	L/R	Brain regions	MNI coordinates			Cluster size	Peak t-value
			*X*	*Y*	*Z*		
**SAs < HCs**							
1	R	Superior temporal gyrus	28.5	19.5	− 43.5	398	− 5.330
2	R	Posterior lobe of cerebellum	24	− 85.5	48	342	− 5.451
3	R	Anterior lobe of cerebellum	28.5	− 43.5	− 36	1482	− 7.345
4	R	Parahippocampal gyrus	33	0	− 25.5	331	− 5.683
5	L	Parahippocampal gyrus	− 18	4.5	− 24	819	− 6.553
6	L	Anterior cingulate	− 10.5	42	16.5	318	− 5.596

The statistical threshold was set at voxel level with *p* < 0.01 for multiple comparisons using Gaussian random field theory voxels with *p* < 0.01.

*GMV* gray matter volume, *SA* strabismus with amblyopia, *HC* healthy control, *MNI* Montreal Neurological Institute, *R* right, *L* left.

**Table 3:** Comparison of whole brain volume between SA and HC group.

	GMV	WMV
SAs	656.02 ± 93.84	479.64 ± 68.52
HCs	677.33 ± 84.91	490.77 ± 46.48
*t* value	− 0.853	− 0.761
*p* value	0.344	0.450

*SA* strabismus with amblyopia, *HC* healthy control, *GMV* gray matter volume, *WMV* white matter volume.

### Correlation analysis

**Figure 2:**
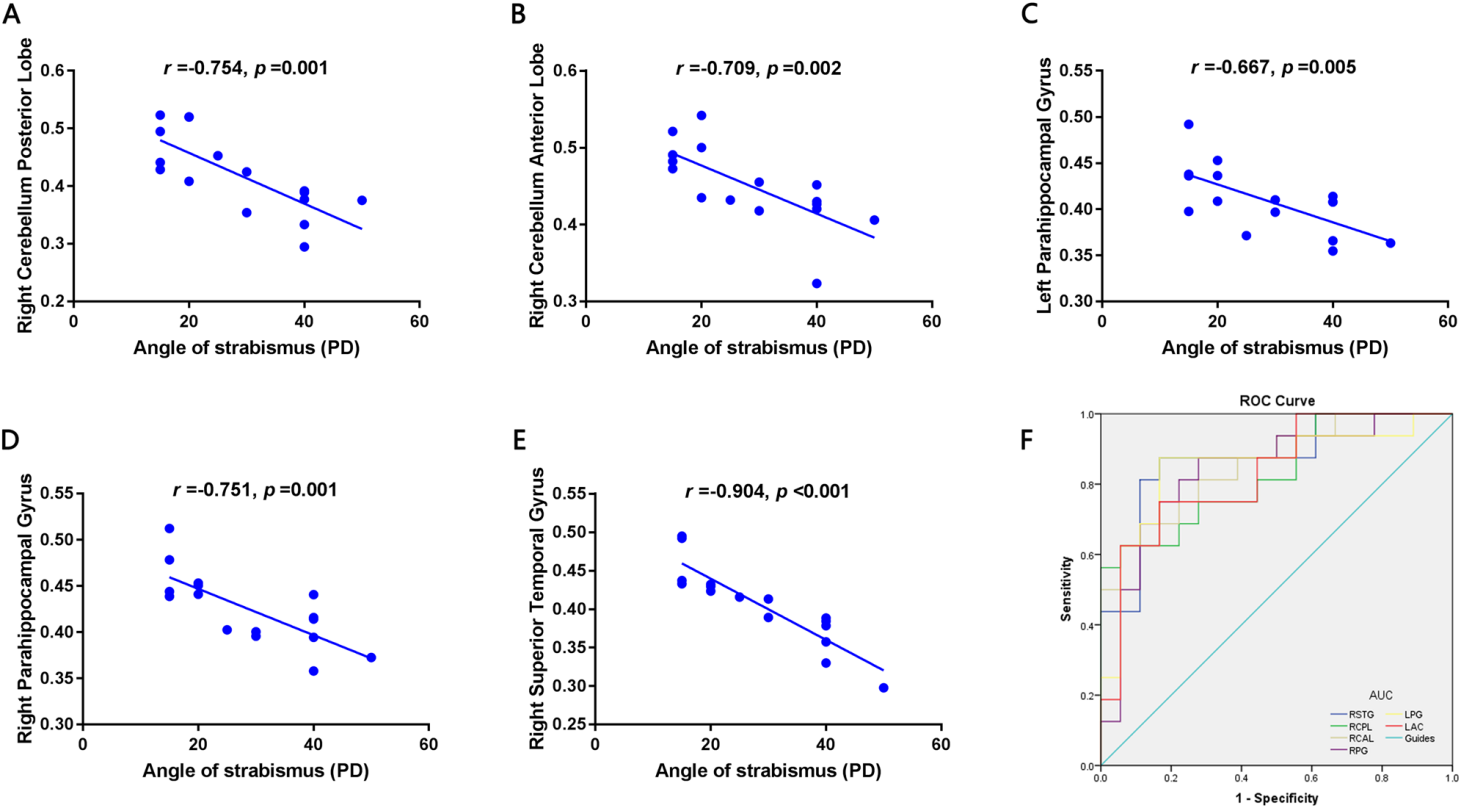
Correlations and ROC curve analysis of the mean GMV values in different regions and the clinical behaviors in SA group. *Notes*: (**A**–**E**) The angle of strabismus showed a negative correlation with the GMV values of the right posterior lobe of cerebrum (*r* = − 0.754, *p* = 0.001), right anterior lobe of cerebrum (*r* = − 0.709, *p* = 0.002), left parahippocampal gyrus (*r* = − 0.667, *p* = 0.005), right parahippocampal gyrus (*r* = − 0.751, *p* = 0.001), right superior temporal gyrus (*r* = − 0.904, *p* < 0.001). (**F**) The area under the ROC curve were 0.872, (*P* < 0.001; 95% CI 0.748–0.995) for RSTG, RCPL 0.830 (*p* = 0.001; 95% CI 0.692–0.968), RCAL 0.854 (*p* < 0.001; 95% CI 0.729–0.980), RPG 0.837 (*p* = 0.001; 95% CI 0.696–0.978), LPG 0.851 (*p* < 0.001; 95% CI 0.710–0.991), LAC 0.830 (*p* = 0.001; 95% CI 0.691–0.969). *GMV* gray matter volume, *SA* strabismus with amblyopia, *PD*, prism diopter, *ROC* receiver operating characteristic, *RSTG* right superior temporal gyrus, *RCPL* posterior lobe of right cerebellum, *RCAL* right anterior lobe of cerebellum, *RPG* right parahippocampal gyrus, *LPG* left parahippocampal gyrus, *LAC* left anterior cingulate.

In the SA group, the angle of strabismus showed a negative correlation with the GMV values of the right posterior lobe of the cerebellum (*r* = − 0.754, *p* = 0.001) (Fig. 2A), right anterior lobe of the cerebellum (*r* = − 0.709, *p* = 0.002) (Fig. 2B), left parahippocampal gyrus (*r* = − 0.667, *p* = 0.005) (Fig. 2C), right parahippocampal gyrus (*r* = − 0.751, *p* = 0.001) (Fig. 2D), and right superior temporal gyrus (*r* = − 0.904, *p* < 0.001) (Fig. 2E).

### ROC curve

In order to verify the hypothesis that the differences in GMV might be possible diagnostic biomarkers to discriminate between the SA and HC groups, the mean GMV values of the various cerebral regions were obtained and subjected to ROC curve analysis. The area under the curve (AUC) of GMV values in each region were as follows: right superior temporal gyrus (0.872, 95% CI 0.748–0.995, *p* < 0.001); right posterior lobe of the cerebellum (0.830, 95% CI 0.692–0.968, *p* = 0.001); right anterior lobe of the cerebellum (0.854, 95% CI 0.729–0.980, *p* < 0.001); right parahippocampal gyrus (0.837, 95% CI 0.696–0.978, *p* = 0.001); left parahippocampal gyrus (0.851, 95% CI 0.710–0.991, *p* < 0.001); and left anterior cingulate cortex (0.830, 95% CI 0.691–0.969, *p* = 0.001) (Fig. 2F). The likelihood ratios and Youden index of each areas are shown in Table 4.

**Table 4:** ROC analysis of the GMV values in different regions between SA and HC groups.

Brain regions	Youden Index	LR+	LR−
Right superior temporal gyrus	0.702	7.324	0.210
Right posterior lobe of cerebellum	0.569	11.161	0.397
Right anterior lobe of cerebellum	0.569	11.161	0.397
Right parahippocampal gyrus	0.597	3.147	0.173
Left parahippocampal gyrus	0.708	5.240	0.150
Left anterior cingulate	0.583	4.491	0.300

*ROC* receiver operating characteristic, *GMV* gray matter volume, *SA* strabismus with amblyopia, *HC* healthy control, *LR*+ positive likelihood ratio, *LR−* negative likelihood ratio.

## Discussion

**Figure 3:**
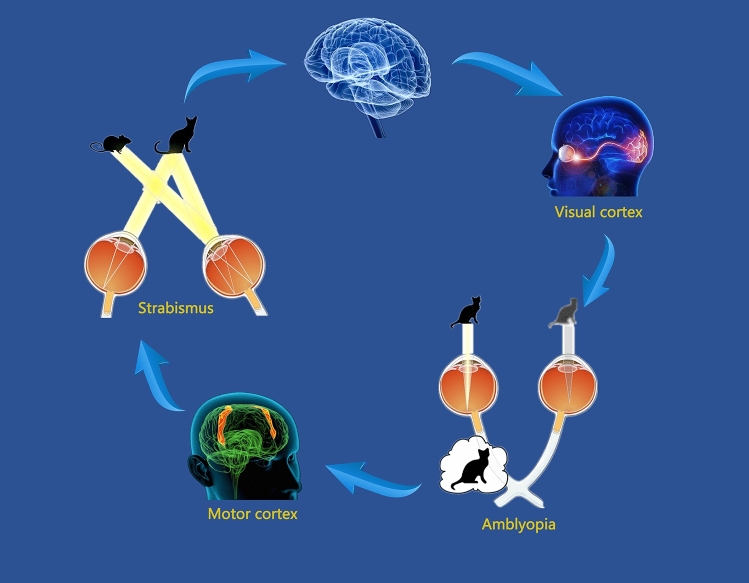
Relationship between MRI images and strabismus with amblyopia. *Notes*: Amblyopia is strongly correlated with the presence of strabismus during early childhood. Early aberrant visual experience could obstruct interocular alignment and ocular movement, leading to strabismus, and disturbing the sensory and visual cortex development might result in amblyopia.

Strabismus and amblyopia are common visual developmental diseases. Their underlying pathogenesis and the relationship and interaction between these two conditions have thus attracted much attention. Previous reports have illustrated that early aberrant visual experience could obstruct interocular alignment and ocular movement, thereby leading to strabismus^19^. In addition, disturbance of the development of the sensory and visual cortex might result in amblyopia^20^. Therefore, the presence of either one of these disorders in early childhood could probably lead to the other (Fig. 3).

Analyses based on VBM constitute a reliable MRI method that can reveal cortical alterations and explore morphological changes in gray matter and white matter in the brain. Previous studies have applied the technique in other ophthalmological diseases (Table 5). To our best knowledge, the present research is the very first to apply the VBM method to patients with SA.

**Table 5:** VBM method applied in ophthalmological diseases.

References	Year	Disease	Brain areas	
			ODs > HCs	ODs < HCs
Xiao et al.^17^	2007	Amblyopia	–	L MFG, L ITG, L parahippocampal gyrus, L fusiform gyrus, B calcarine cortices
Chan et al.^4^	2004	Strabismus	FEF, SEF, PFC, thalamus	OEF, PEF
Li et al.^12^	2012	Primary open angle glaucoma	BA39	B PVC, R MFG, R ITG, B paracentral lobule, R precentral gyrus, R angular gyrus, L precuneus, L middle temporal gyrus, superior temporal gyrus
Huang et al.^13^	2016	Optic neuritis	-	B MFG, L postcentral gyrus, L inferior frontal gyrus, L anterior cingulate, R inferior parietal lobule
Hou et al.^39^	2017	Blindness	L lateral middle occipital gyri	L lateral calcarine cortices

*VBM* voxel-based morphometry, *OD* ophthalmological disease, *HC* healthy control, *L* left, *R* right, *B* bilateral, *MFG* left middle frontal gyrus, *ITG* inferior temporal gyrus, *OEF* occipital eye field, *PEF* parietal eye field, *FEF* frontal eye field, *SEF* supplementary eye field, *PFC* prefrontal cortex, *PVC* primary visual cortex, *BA* Brodmann area.

**Figure 4:**
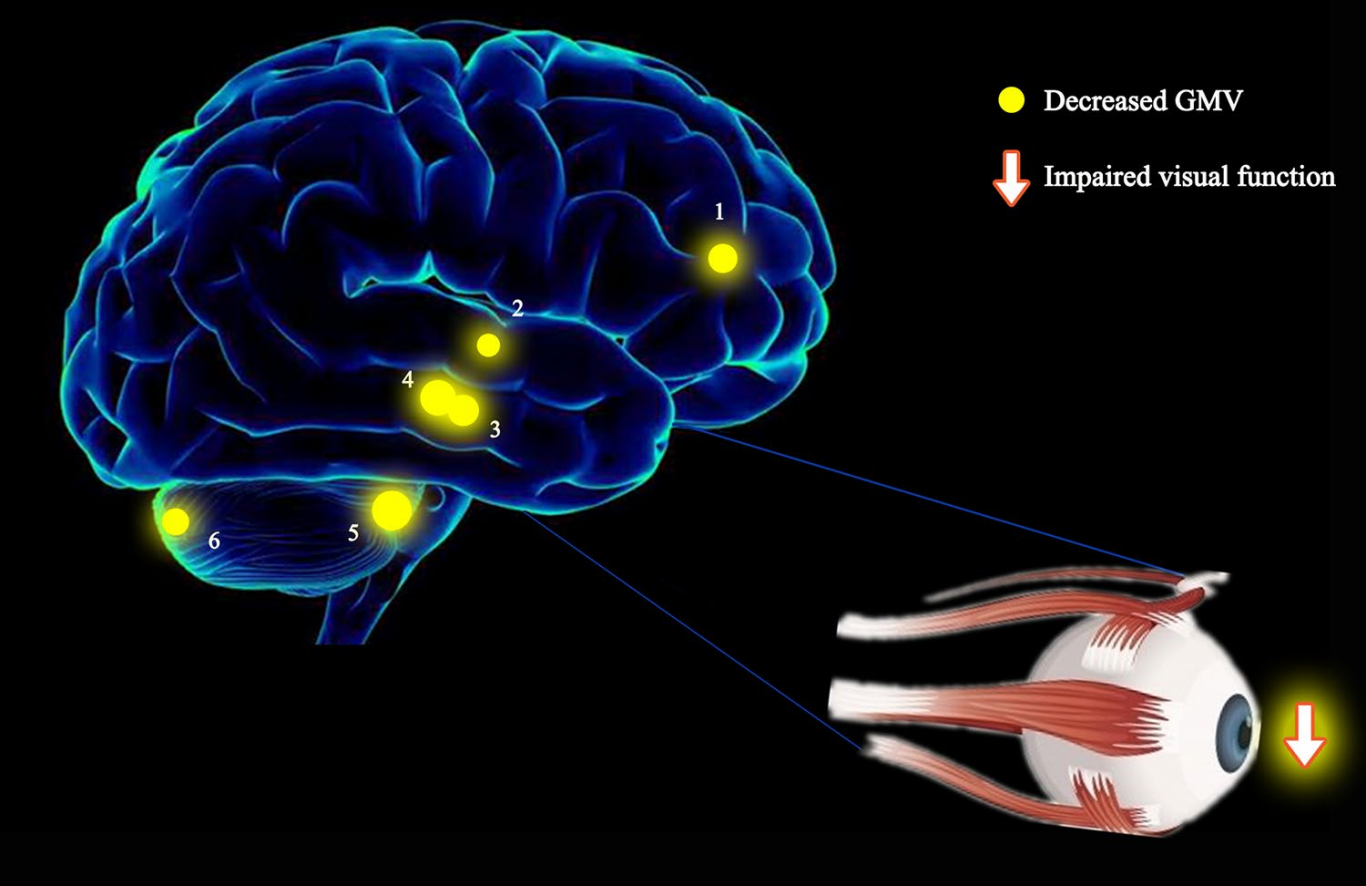
The GMV results of brain activity in the SA group. Compared with the HCs, the GMV of SA patients in the following regions were decreased to various extents: 1-left anterior cingulate (t = − 5.596), 2-right superior temporal gyrus (t = − 5.330), 3-right parahippocampal gyrus (t = − 5.683), 4-left parahippocampal gyrus (t = − 6.553), 5-right anterior lobe of cerebellum (t =  − 7.345), 6-right posterior lobe of cerebellum (t = − 5.451). *Notes*: The sizes of the spots denote the degree of quantitative changes. *GMV* gray matter volume, *SA* strabismus with amblyopia, *HC* healthy control.

In this study, we discovered a significantly reduced GMV in the right superior temporal gyrus, posterior and anterior lobes of the cerebellum, bilateral parahippocampal gyrus, and left anterior cingulate cortex, with impaired visual function in patients with SA (Fig. 4).

The cerebellum is an isolated structure at the bottom of the cerebrum, tucked beneath the cerebral hemispheres. Previous studies have reported that the cerebellum is involved in motion control and perception^21^, especially the execution of ocular movements^22–24^ and visually guided saccades^25^. Ouyang et al. reported that individuals with concomitant strabismus had declining GMV in the left posterior lobe of the cerebellum^16^. Studies on individuals with concomitant exotropia have shown that the DC value and mean diffusivity value in the right posterior lobe of the cerebellum were markedly reduced^26,27^. Furthermore, our previous research on patients with SA demonstrated that the ALFF values in the left posterior lobe of the cerebellum were reduced, when compared with HCs^10^. Similar alterations were observed in subjects with anisometropic amblyopia and reduced spontaneous activity in the cerebellum^28^. In line with previous reports, we also observed that subjects with SA showed significantly lower GMV values in the right posterior lobe and anterior lobe of the cerebellum, indicating damage associated with the execution of eye movement in this region.

The superior temporal gyrus lies laterally to the cerebrum, and is located in a superior position to the external ear. It is implicated in auditory processing, language comprehension, as well as social cognition^29,30^. Recent investigations on the function of the superior temporal gyrus have uncovered its relationship with eye movement processing and visual analyses of social information transmitted by gaze^11,31^. Prior studies have reported inconsistent results in this area of the brain. Some have demonstrated that adults with concomitant exotropia exhibit increased degree centrality values of the right superior temporal gyrus^26^. Several investigations have focused on subjects with anisometropic amblyopia, who also showed increased spontaneous brain activity in the superior temporal gyrus^28,32^. Nevertheless, Huang et al. reported significantly reduced fractional anisotropy values in the superior temporal gyrus of subjects with concomitant strabismus^27^. Moreover, patients with primary open-angle glaucoma displayed reduced GM density, mainly in the left superior temporal gyrus^12^. In the present study, the subjects with SA showed decreased GMV values in the right superior temporal gyrus, which further corroborates the notion that SA may lead to dysfunction of the temporal gyrus.

The parahippocampal gyrus is a GM cortical area of the brain that encircles the hippocampus, and plays a major role in memory encoding and retrieval. A previous report found reduced GMV values in the bilateral parahippocampal gyrus of patients with monocular amblyopia^15^. Similarly, reduced gray matter density of the parahippocampal gyrus was detected in children with amblyopia^17^. In addition, Berberat and associates also proved that images of strabismus could induce activation of the parahippocampal gyrus, when compared with images of normal eyes^33^. Consistent with these findings, the reduced GMV value of the bilateral parahippocampal gyrus in the present study may indicate injury in patients with SA.

The cingulate gyrus is an essential portion of the limbic system, which plays a role in the formation of emotions^34^, depression^35^, and pain^36^. The limbic system is closely associated with memory and emotion^37^. Previous studies about patients with concomitant strabismus have shown activation in the region of the anterior cingulate cortex^27,38^. Adult subjects with concomitant esotropia also evidently exhibit increased voxel-wise degree centrality values in the bilateral anterior cingulate cortex^26^. Moreover, Chan et al. showed that adults with strabismus exhibit greater gray matter volume in the anterior cingulate gyrus^4^. However, reduced GMV was observed in the left anterior cingulate cortex of individuals with optic neuritis^13^. In the present study, GMV reduction might have been attributed mainly to the poor vision associated with amblyopia, rather than strabismus.

Correlation analysis revealed that the angle of strabismus in SA is negatively correlated with the mean GMV values of several brain regions (Fig. 2). A greater angle of strabismus indicated more severe SA. Thus, we might presume that more severe strabismus can lead to greater atrophy of the gray matter. Accordingly, we suspect the reduction in GMV in those cerebral areas may be the underlying pathological mechanism of ocular motor disorders in patients with SA.

The ROC curve analyses were used to distinguish diseased individuals from HCs, with high sensitivity and specificity (Fig. 2F). When the AUC was over 0.8, it denoted perfect accuracy; an AUC between 0.6 and 0.8 meant the accuracy was moderate; and if the AUC was less than 0.6, diagnostic value was limited. In the present study, ROC curve analysis revealed that perfect AUC values were observed among all areas of interest, including the superior temporal gyrus, posterior and anterior lobes of the cerebellum, parahippocampal gyrus, and anterior cingulate cortex, indicating that the VBM method could be useful in characterizing the neural mechanisms underlying SA, and may be capable of detecting early biomarkers of SA.

Nevertheless, some limitations in the present research need to be considered. For instance, a relatively insufficient number of participants were enrolled, which may have affected reliability. In addition, various types of strabismus were implicated, which are supposed to be categorized in a subsequent study, to probe the structural alterations of the brain more precisely.

## Conclusions

VBM is a computational approach to neuroanatomy that allows for comprehensive measurement of cerebral differences, not just in specific structures with traditional morphometry, but throughout the entire brain. In this study, we found changes not only in the visual-related and motion-related brain regions, which we could predict, but also in emotion-related area (cingulate gyrus). These findings might help to reveal the potential pathogenesis of SA and its relationship with atrophy in specific regions of the brain.
